# Outer membrane tube formation by *Francisella novicida* involves extensive envelope modifications and is linked with type VI secretion and alterations to the host phagosomal membrane

**DOI:** 10.1128/mbio.01060-25

**Published:** 2025-05-19

**Authors:** Maheen Rashid, Shoichi Tachiyama, Shiwei Zhu, Hang Zhao, William D. McCaig, Jingchuan Sun, Huilin Li, Jun Liu, David G. Thanassi

**Affiliations:** 1 Department of Microbiology and Immunology, Renaissance School of Medicine, Stony Brook University 12301, Stony Brook, New York, USA; 2 Center for Infectious Diseases, Stony Brook University 12301, Stony Brook, New York, USA; 3 Department of Microbial Pathogenesis, Yale School of Medicine 12228 , New Haven, Connecticut, USA; 4 Microbial Sciences Institute, Yale University 5755, New Haven, Connecticut, USA; 5 Department of Structural Biology, Van Andel Institute 3584, Grand Rapids, Michigan, USA; University of Michigan-Ann Arbor, Ann Arbor, Michigan, USA

**Keywords:** *Francisella*, outer membrane vesicles, bacterial pathogens, type VI secretion system, macrophage infection

## Abstract

*Francisella tularensis* is a gram-negative, intracellular pathogen that causes the zoonotic disease tularemia. Due to its ease of dissemination and high lethality, *F. tularensis* is classified as a tier 1 select agent with potential for misuse as a bioweapon. The mechanisms by which *Francisella* replicates intracellularly and interacts with the host during infection are not well understood. *Francisella* produces spherical outer membrane vesicles (OMVs) and novel tubular extensions of its cell surface that are also released extracellularly. These OMV and outer membrane tubes (OMTs) contain *Francisella* virulence factors and are produced in response to amino acid starvation and during infection of macrophages. To investigate how the OMTs are formed, we used cryogenic electron tomography to examine the model *Francisella* spp., *Francisella novicida*, during *in vitro* culture and within the macrophage phagosome. OMT formation involved progressive alterations of the bacterial envelope, resulting in extensions of both the inner and outer membranes. A dynamic cytoplasmic structure was present at the base of the OMT that extended into the tubes during elongation, together with cytoplasmic material. OMT produced within the macrophage phagosome was associated with changes to the phagosomal membrane, suggesting a role in phagosomal escape. Consistent with this, using confocal microscopy, we observed co-localization of the *Francisella* type VI secretion system with the OMT, both within bacteria and in released tubular vesicles. These findings reveal the cellular transformations that occur during membrane tubulation by *Francisella* and provide insights into the function of membrane-derived structures during host-pathogen interactions.

**IMPORTANCE:**

*Francisella tularensis* is an intracellular bacterial pathogen that causes the zoonotic disease tularemia. Following uptake by host cells, the bacteria rapidly escape the phagosome and replicate intracellularly. In previous studies, we found that *Francisella* produces tubular extensions of its cell surface in response to specific cues and during macrophage infection. In the present study, we used cryogenic electron tomography to examine tube formation by the model *Francisella* sp., *F. novicida*. This analysis revealed that tube formation involves extensive bacterial envelope alterations and a dynamic cytoplasmic organelle. Furthermore, tubes produced by bacteria within infected macrophages were associated with the breakdown of the phagosomal membrane. In addition, we found that the *Francisella* type VI secretion system, which is essential for phagosomal escape, co-localized with the bacterial tubes. These findings reveal the cellular transformations that occur during membrane tubulation by *Francisella* and suggest a role for the tubes in phagosomal escape.

## INTRODUCTION

*Francisella tularensis* is a highly virulent, gram-negative, facultative intracellular bacterium and causative agent of the zoonotic disease tularemia (1–4). Inhaling even a very small dose of aerosolized bacteria leads to severe pneumonia, with a mortality rate as high as 60% if left untreated (5). Due to its low infectious dose, ease of aerosolization, and potential for high morbidity and mortality, *F. tularensis* is categorized by the Centers for Disease Control and Prevention as a tier 1 select agent (6). There are two subspecies of *F. tularensis* with clinical significance: subsp. *tularensis* and *holarctica* (6). Another species within the *Francisella* genus, *Francisella novicida*, has limited virulence in healthy humans while maintaining its capability to cause severe diseases in animal models. *F. novicida* has served as an important experimental strain that can be handled under biosafety level 2 conditions (7). The molecular basis for *Francisella*’s virulence and extreme infectivity remains poorly understood. This, together with the absence of a licensed and effective vaccine, makes it important to understand the mechanisms of *F. tularensis* intracellular pathogenesis to support the advancement of therapeutic strategies against tularemia.

*Francisella* infects and replicates within a variety of host cells, with macrophages being a preferred intracellular niche (6, 8, 9). Following internalization, *Francisella* is initially contained within the macrophage phagosome. The bacteria suppress maturation of the phagosome, avoid or interfere with intracellular signaling pathways, and resist host defense mechanisms (2, 3, 10, 11). *Francisella* rapidly escapes from the phagosome and enters the host cell cytosol, where bacterial replication occurs. Extensive replication of the bacteria eventually results in host cell death and bacterial release for subsequent rounds of infection (8, 12, 13). Critical to *Francisella*’s ability to escape the phagosome is a genomic locus termed the *Francisella* pathogenicity island (FPI) (8, 12, 14–16). The FPI contains genes encoding a type VI secretion system (T6SS), which is essential for phagosomal escape (14, 15, 17, 18). *Francisella* spp. also encode a type I secretion system and type IV pilus assembly pathway, which are linked to effector secretion (19–21). Given its high pathogenicity and ability to replicate intracellularly while suppressing host responses, it is likely that *Francisella* utilizes additional pathways for virulence factor secretion.

Previous work demonstrated the production of outer membrane vesicles (OMVs) by *Francisella* spp. (22–25). OMV has been shown to deliver effector proteins to host cells and modulate host immune responses, supporting a role for OMV as a virulence factor secretion system (26, 27). In addition to spherical OMV, *Francisella* spp. produce unique tubular vesicles and tubular extensions of its cell surface, termed outer membrane tubes (OMTs) (23–25). The OMTs are produced during infection of macrophages, including within the macrophage phagosome, suggesting they function during intracellular pathogenesis (8, 23, 28). In previous studies, we demonstrated that *F. novicida* produces OMT in a controlled manner that is regulated by specific genes and environmental signals (23, 25). We identified the inducing signal as amino acid starvation, including cysteine deprivation (25). Amino acid starvation is experienced by the bacteria during infection of macrophages and is also a signal used to turn on genes within the FPI, including the T6SS (29–31).

In this study, we employed cryogenic electron tomography (cryo-ET) to conduct a detailed analysis of the *F. novicida* OMT. Bacterial OMT formation occurred at the cell pole and involved extension of both the inner membrane (IM) and outer membrane (OM). We detected a dynamic cytoplasmic structure positioned at the base of the tubes. This structure changed shape and extended into the growing tubes, together with cytoplasmic material and the IM. Imaging of released OMT revealed that the released tubes also contained cytoplasmic and IM material. We used cryo-focused ion beam milling coupled with cryo-ET (cryo-FIB-ET) to visualize OMT produced by *F. novicida* inside the macrophage phagosome. Notably, possible alterations in the phagosomal membrane occurred at contact points between the OMT and the phagosome, suggesting a role in phagosomal escape. Consistent with this, using confocal microscopy, we detected co-localization of the T6SS with both bacterial and released OMT. These findings provide evidence for a sophisticated mechanism governing the generation of OMT and reveal their association with a key step in the intracellular pathogenesis of *Francisella*.

## RESULTS

### Cryo-ET imaging of *F. novicida* reveals changes to the bacterial envelope upon tube formation and a novel cytoplasmic structure

**Fig 1:**
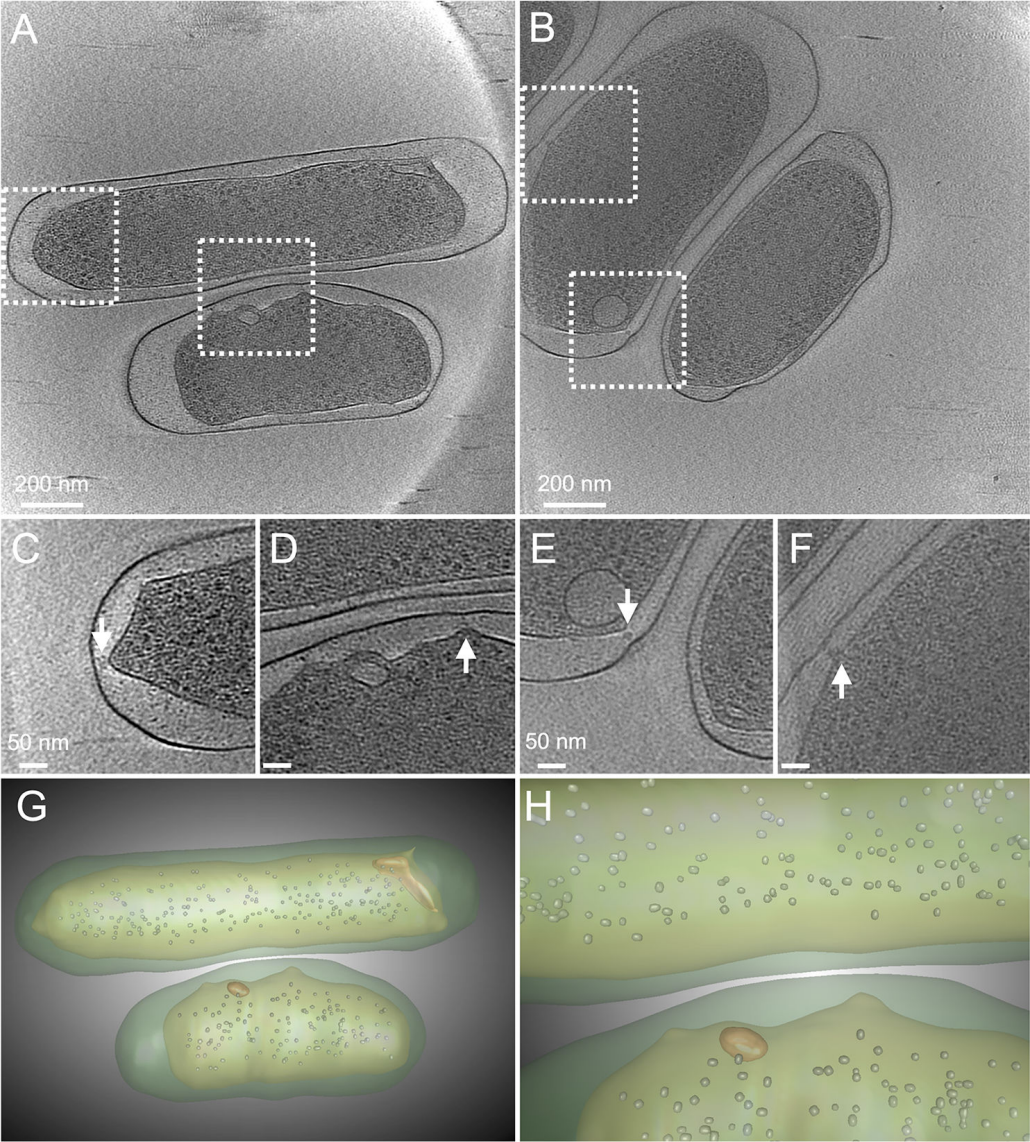
Cryo-ET imaging of *F. novicida* grown under cysteine-sufficient (OMT-repressing) conditions. (A and B) Single sections of tomograms showing *F. novicida* U112 grown on BHI agar with 0.1% cysteine supplementation. (C–F) Zoomed-in images of boxed regions in panels A and B highlighting oval-shaped cytoplasmic and periplasmic structures that bridge the IM and OM (arrows). The image in panel C corresponds to the upper left boxed region in panel A but from a different section of the same tomogram (see Movie S1). (G and H) Overview (**G**) and zoomed-in (**H**) segmentation images of the tomogram shown in panel A. The cytoplasmic structures are shown in orange, and ribosomes (small round shapes) are shown in gray. The cytoplasm and IM are indicated by light green, and the periplasm and OM are indicated by dark green. The images shown are representative of the nine bacteria analyzed.

We first used cryo-ET to examine *F. novicida* U112 grown under conditions in which the bacteria do not experience amino acid starvation and OMT production is not induced. The bacteria were grown for 44–48 h on brain heart infusion (BHI) agar supplemented with 0.1% cysteine. *F. novicida* grown under these conditions exhibited the characteristic appearance of gram-negative bacteria, with a relatively uniform coccobacillus shape (Fig. 1A and B; Movies S1 and S2). The bacteria contained a typical, dense cytoplasm populated with ribosomes, surrounded by a distinct IM, periplasm, and OM. The periplasm was mostly thin and evenly spaced. However, enlarged periplasmic regions were present, especially at the cell poles (Fig. 1A and B), and unevenness in the IM and OM was apparent, together with periplasmic structures of unknown identity that appeared to bridge between the membranes (Fig. 1C through F). Notably, oval-shaped structures were observed within the cytoplasm of the bacteria, positioned adjacent to the IM and located at or near a cell pole (Fig. 1A, B, D, and E; Movies S1 and S2). These oval cytoplasmic structures measured ~50 to 125 nm in diameter and appear to be membrane bound, with a clearly defined border and lighter interior density than the surrounding cytoplasm. In some bacteria, two such cytoplasmic structures were present (Movie S2), and we also observed a more extended cytoplasmic structure in one bacterium (Fig. 1A and G).

**Fig 2:**
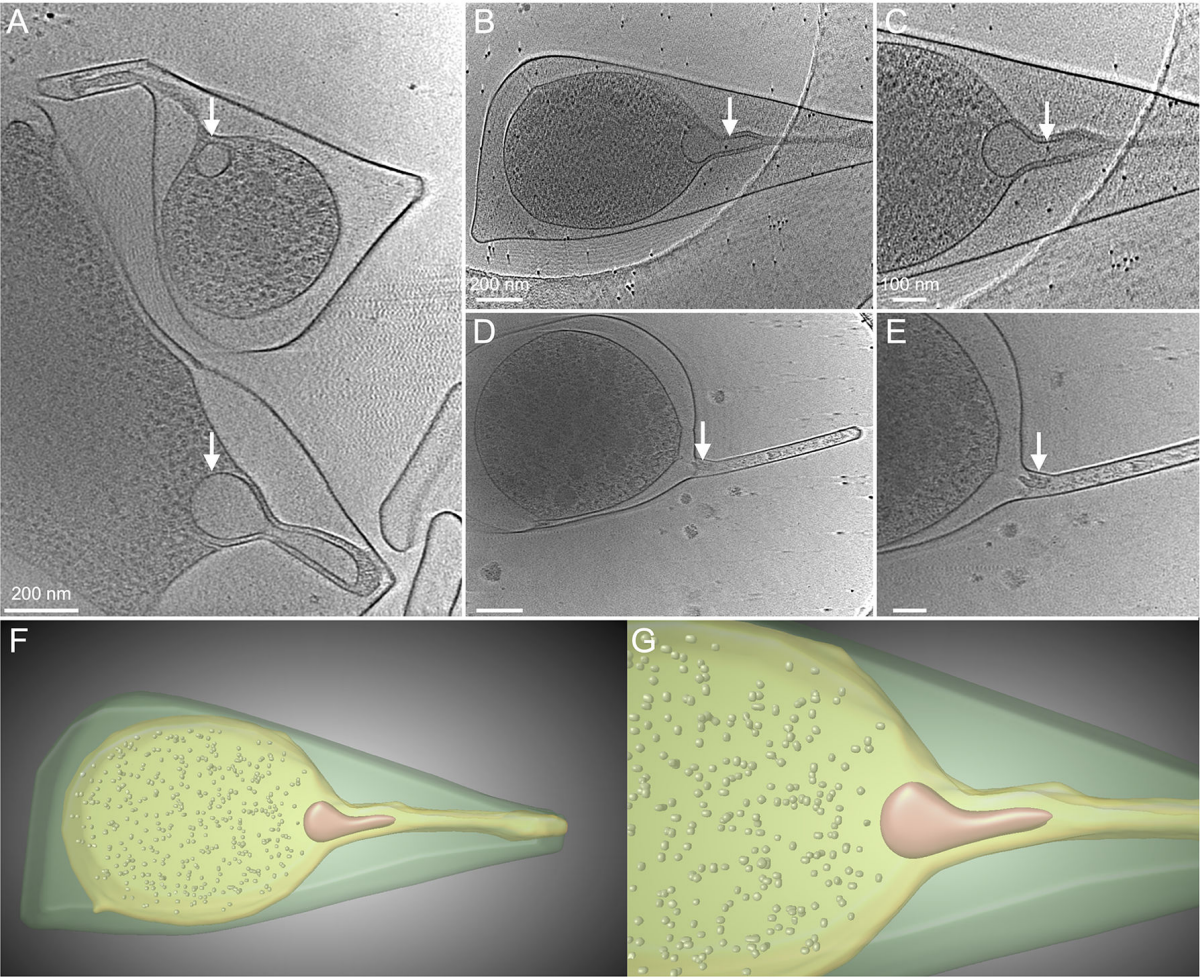
Cryo-ET imaging of *F. novicida* grown under OMT-inducing conditions. (A, B, and D) Single sections of tomograms showing *F. novicida* U112 grown on BHI agar without cysteine supplementation. (C and E) Zoomed-in images of panels B and D. (B and C) The cytoplasmic bulb-like structure (arrows) extending into the OM, along with cytoplasmic material. (D and E) A narrower OMT, with aggregated material present inside the tube (arrow) and a fragmented cytoplasmic structure. (F and G) Segmentation images of the tomogram corresponding to the images in panels B and C. The bulb-like structure and ribosomes are shown in orange and gray, respectively. The cytoplasm and IM are indicated by light green, and the periplasm and OM are indicated by dark green. The images shown are representative of the 14 bacteria analyzed.

We next examined *F. novicida* U112 grown for 44–48 h on BHI agar without cysteine supplementation. Under these conditions, the bacteria deplete the available cysteine, leading to amino acid starvation and the induction of OMT (25). Bacteria grown in these conditions exhibited substantial changes to their cell envelope and overall shape (Fig. 2; Movies S3 to S5). The bacteria became pleomorphic, with a more rounded cytoplasm and prominent bulges and distended regions of the periplasm and OM. Multiple extended regions of the OM were observed per bacterium, typically at the cell poles (Fig. 2A through E). In some cases, one of these regions extended further to create a tubular projection from the bacterial cell surface (Fig. 2A, B, and D; Movies S3 to S5). Dense structures were visible in the cytoplasm of some bacteria (Fig. 2D and E), which may represent the formation of stress granules under the amino acid starvation conditions (32, 33). The previously observed oval cytoplasmic structures remained present in the bacteria but underwent significant changes, enlarging and transforming into a more bulb-like conformation, the tip of which extended into the OMT (Fig. 2A through C, F, and G; Movies S3 to S5). The bulb-like structure appeared to form the base or origin point of the OMT, with the IM and associated cytoplasmic material extending further into the tube. In some cases, the cytoplasmic structure at the base of the OMT appeared to be fragmented (Fig. 2D and E; Movie S5). In these cases, the periplasm was less enlarged, and the OMT was narrower, with condensed or aggregated material present inside the tube (Fig. 2D and E; Movie S5).

**Fig 3:**
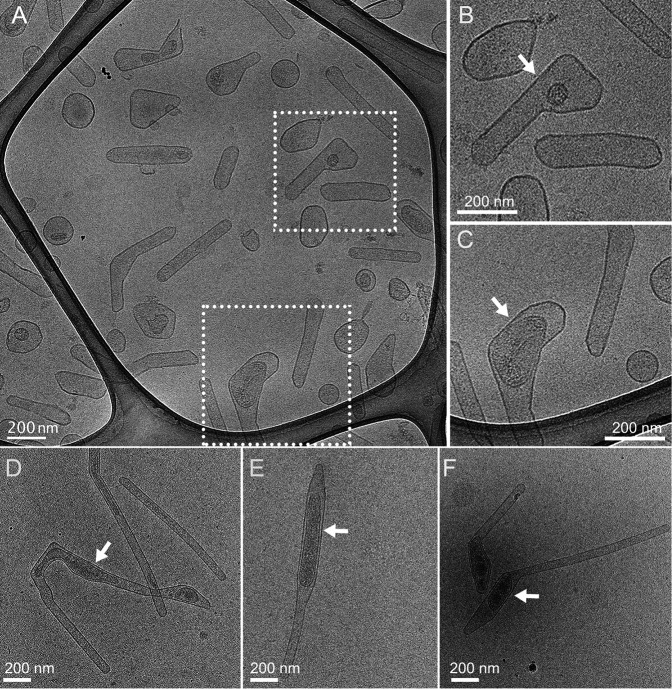
Cryo-EM imaging of vesicles released by *F. novicida* into the surrounding medium. (A and D–F) Representative cryo-EM images of vesicles released by *F. novicida* U112 grown on BHI agar without cysteine supplementation. (B and C) Enlarged views of the boxed regions in panel A. The arrows point to internal structures within the released OMT.

In addition to extending from the bacterial surface, the *Francisella* OMT is released into the surrounding medium, together with spherical OMVs (23–25). We used cryo-EM to examine vesicles released from *F. novicida* grown under tube-inducing conditions (BHI agar without cysteine supplementation). The vesicles exhibited a range of shapes and sizes, corresponding to both released OMT and OMV (Fig. 3A). Internal structures were evident for many of the vesicles, with density and appearance consistent with cytoplasmic material surrounded by an IM (Fig. 3B through F). Thus, the cytoplasmic and IM regions observed extending into tubes on the bacterial surface were maintained within the released OMT.

### Progression of *F. novicida* tube formation upon exposure to amino acid starvation

**Fig 4:**
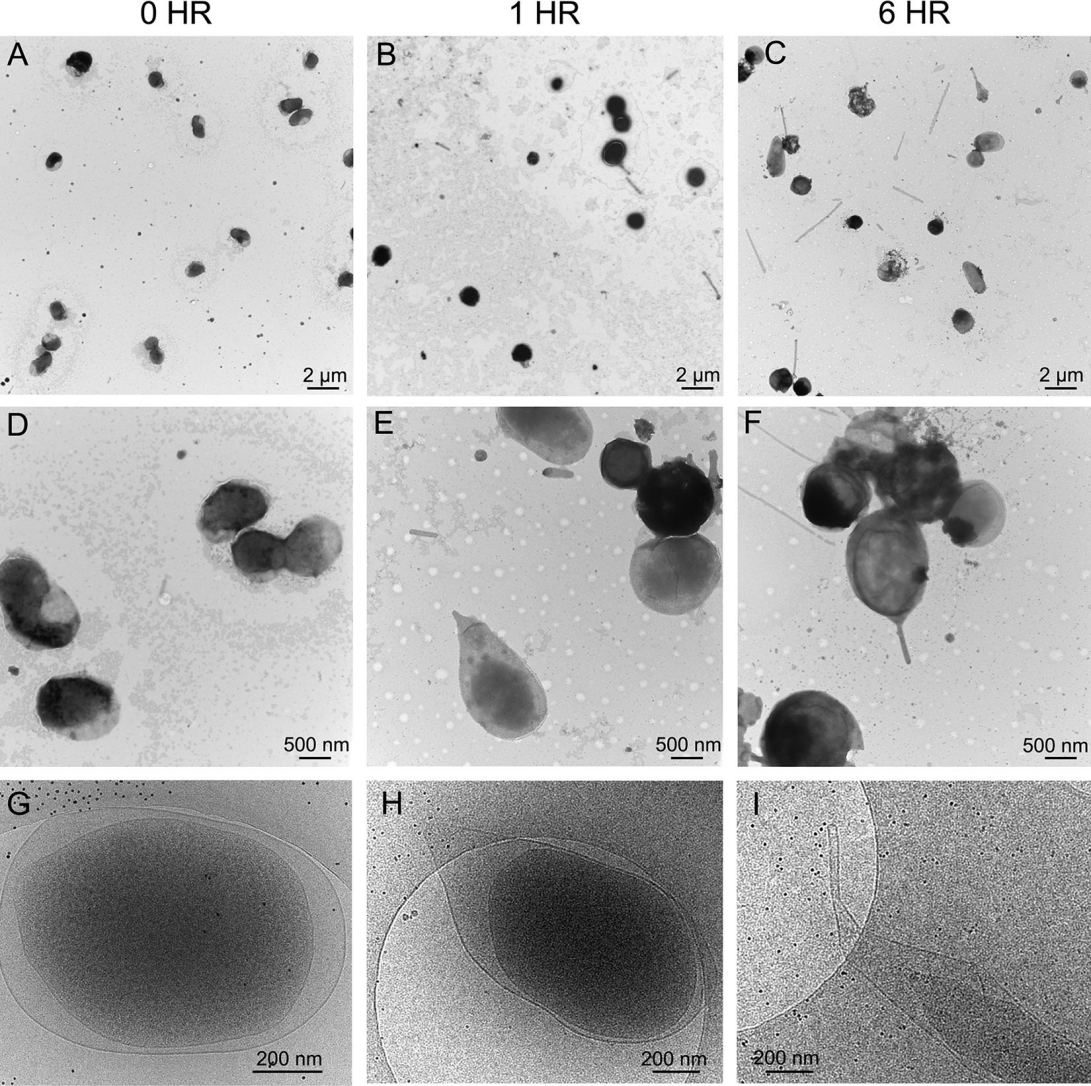
Progression of OMT formation upon exposure of *F. novicida* to amino acid starvation. Representative TEM (A–F) and cryo-EM (G–I) images of *F. novicida* U112 grown to early log phase in BHI medium and then resuspended into conditioned BHI medium depleted of cysteine. Bacteria were captured just prior to resuspension in the conditioned medium at 0 h time point (A, D, and G), 1 h after resuspension (B, E, and H), and 6 h after resuspension (C, F, and I). Bacteria at 0 h show a regular morphology, with OMT formation increasing from 1 to 6 h. (A–F) Representative images of ~80 bacteria analyzed at each time point. (G–I) Representative images of ~10 bacteria analyzed at each time point.

To investigate the progression of tube formation by *F. novicida*, we developed an assay to synchronize the initiation of the bacterial response to amino acid starvation. *F. novicida* grown in BHI broth to early log phase was harvested and resuspended in cell-free, conditioned BHI medium obtained from stationary-phase *F. novicida* cultures. This conditioned medium was depleted for cysteine, resulting in synchronized exposure of the bacteria to amino acid starvation upon resuspension. Bacteria examined by negative-stain transmission electron microscopy (TEM), just prior to resuspension in the conditioned BHI medium, were uniform in shape and lacked OMT (Fig. 4A, D, and G), similar to the bacteria grown on cysteine-supplemented BHI agar (Fig. 1). Of 76 bacteria imaged by TEM at the 0 h time point, zero bacterial and two released OMTs were detected. By 1 h post-resuspension in the conditioned medium, tube formation had initiated on a subset of the bacteria, and released OMTs were visible on the EM grids (Fig. 4B and E). Of 70 bacteria imaged by TEM at the 1 h time point, 9 bacterial and 19 released OMTs were detected. By cryo-EM, extended regions of the bacterial OM were present at the cell poles, with a corresponding expansion of the periplasm at these sites of protrusion (Fig. 4H). The OM extensions at the 1 h time point were broad at the base and rounded at the tip, reflecting an early stage of tube formation. By 6 h post-resuspension, there was a marked increase in both bacterial and released OMTs compared to the 1 h time point (Fig. 4C and F). Of 86 bacteria imaged by TEM at the 6 h time point, 3 bacterial and 54 released OMTs were detected. By cryo-EM, the bacterial OMT projections were elongated and had a more defined, tubular shape (Fig. 4I). These results show that tube production by *F. novicida* is an early response following the transition from a nutrient-rich to a nutrient-depleted environment and involves progressive restructuring of the OM, with corresponding expansion of the periplasm and changes to the IM and cytoplasm at the base of the tubes.

### *F. novicida* tubes are produced during macrophage infection and co-localize with alterations in the phagosomal membrane

**Fig 5:**
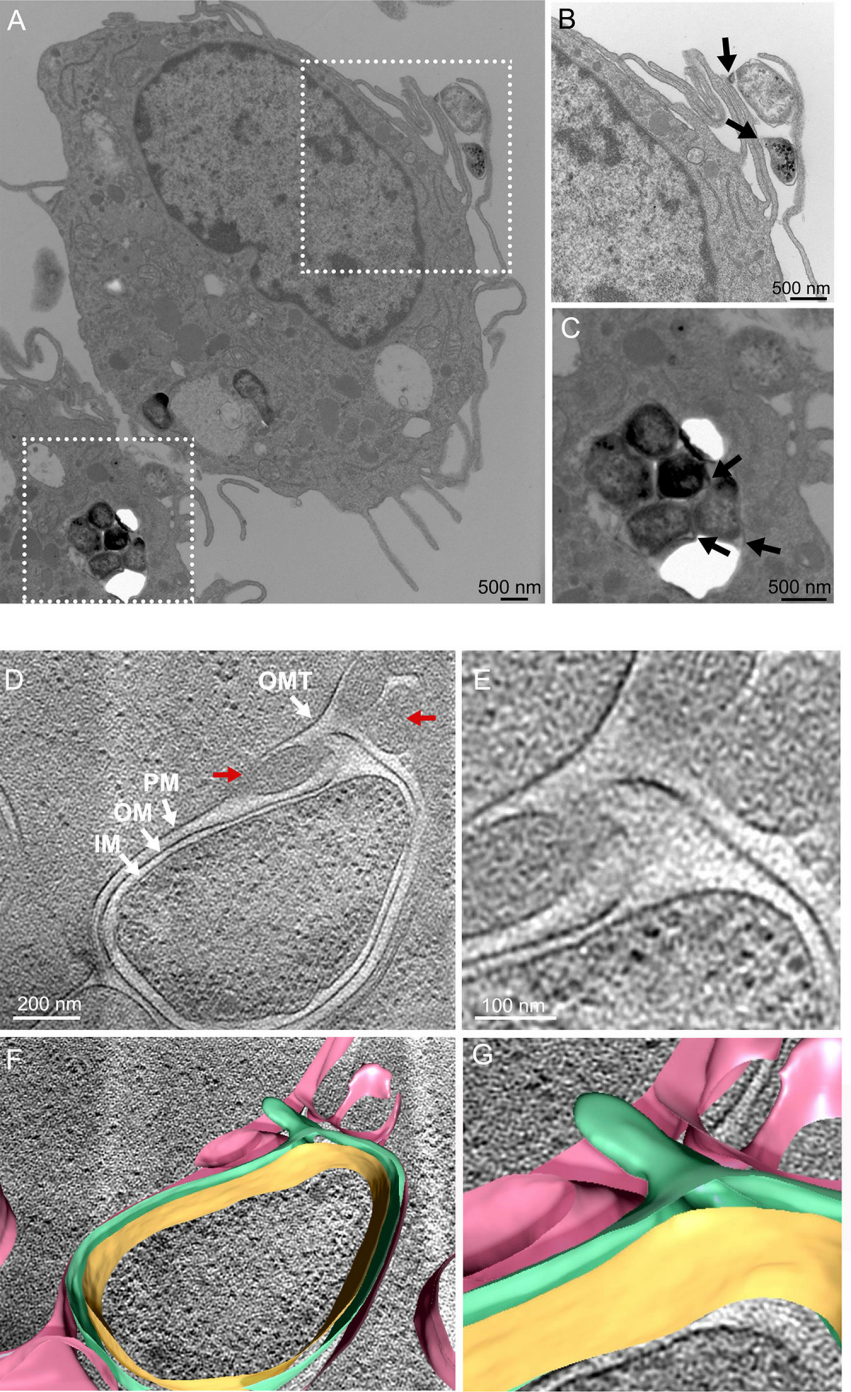
*F. novicida* produces OMT during macrophage infection and triggers phagosomal membrane rearrangement. (A–C) Thin-section TEM image of RAW 264.7 macrophage-like cells at 20 min post-infection with *F. novicida* U112. (B and C) Enlarged views of the boxed regions in panel A. The arrows indicate regions of OMT formation on bacteria at the macrophage cell surface (**B**) or within the phagosome (**C**). (D–G) Single section of a tomogram capturing an intracellular *F. novicida* in the macrophage phagosome. In panel D, the white arrows indicate the bacterial IM, OM, and OMT, and the host cell phagosomal membrane (PM). The red arrows indicate regions surrounding the OMT where the phagosomal membrane is undergoing vesiculation. (E) Zoomed-in image of the OMT region in panel D. (F and G) Overlaid tomogram section and 3D segmented images representing the bacterial IM (orange) and OM (green), and the host cell phagosomal membrane (pink). The images shown in panels A–C are representative of ~50 bacteria analyzed that were intracellular or interacting with the macrophage surface. The image of the intraphagosomal bacterium shown in panel D is representative of two such images captured.

To investigate the production of OMT during host cell infection, RAW 264.7 macrophage-like cells were infected with *F. novicida* U112 at a multiplicity of infection (MOI) of 2,000. At 20 min post-infection, the infected macrophages were processed for thin-section, negative-stain TEM (Fig. 5A through C). The high MOI was used to facilitate detection of the bacteria in the sections, and the early time point was chosen to capture bacteria at the macrophage surface or in the phagosomal compartment. The TEM images confirmed the presence of OM protrusions on bacteria that were interacting at the macrophage surface or located within phagosomes (Fig. 5B and C). The bacteria were irregular in shape and contained distended periplasmic regions, consistent with cultured bacteria grown under tube-inducing conditions (Fig. 2 and 4).

We next used cryo-FIB-ET to obtain a higher-resolution view of *F. novicida* within the RAW 264.7 phagosome in a near-native state. As shown in Fig. 5D through G; Movie S6, we were able to capture *F. novicida* enclosed within the macrophage phagosome. In the cryo-FIB-ET images and corresponding 3D segmentation analysis (Fig. 5F and G; Movie S6), the bacterial cytoplasm, IM, periplasm, and OM were clearly defined, and the phagosomal membrane surrounding the bacterium was also visible. A tubular extension of the OM was seen emerging from one pole of the bacterium, enclosing an enlarged periplasmic region (Fig. 5D through G; Movie S6). Notably, at the position where the tip of the tubular projection extended toward the phagosomal membrane, possible alterations of the phagosomal membrane were present, with the membrane appearing to vesiculate or break into smaller structures, although this could also represent folds in the membrane rather than phagosomal breakdown. Nevertheless, the imaging suggested the possibility that the OMT might be associated with the process of bacterial escape from the phagosome into the cytoplasm.

### The *F. novicida* T6SS co-localizes with bacterial and released OMTs

**Fig 6:**
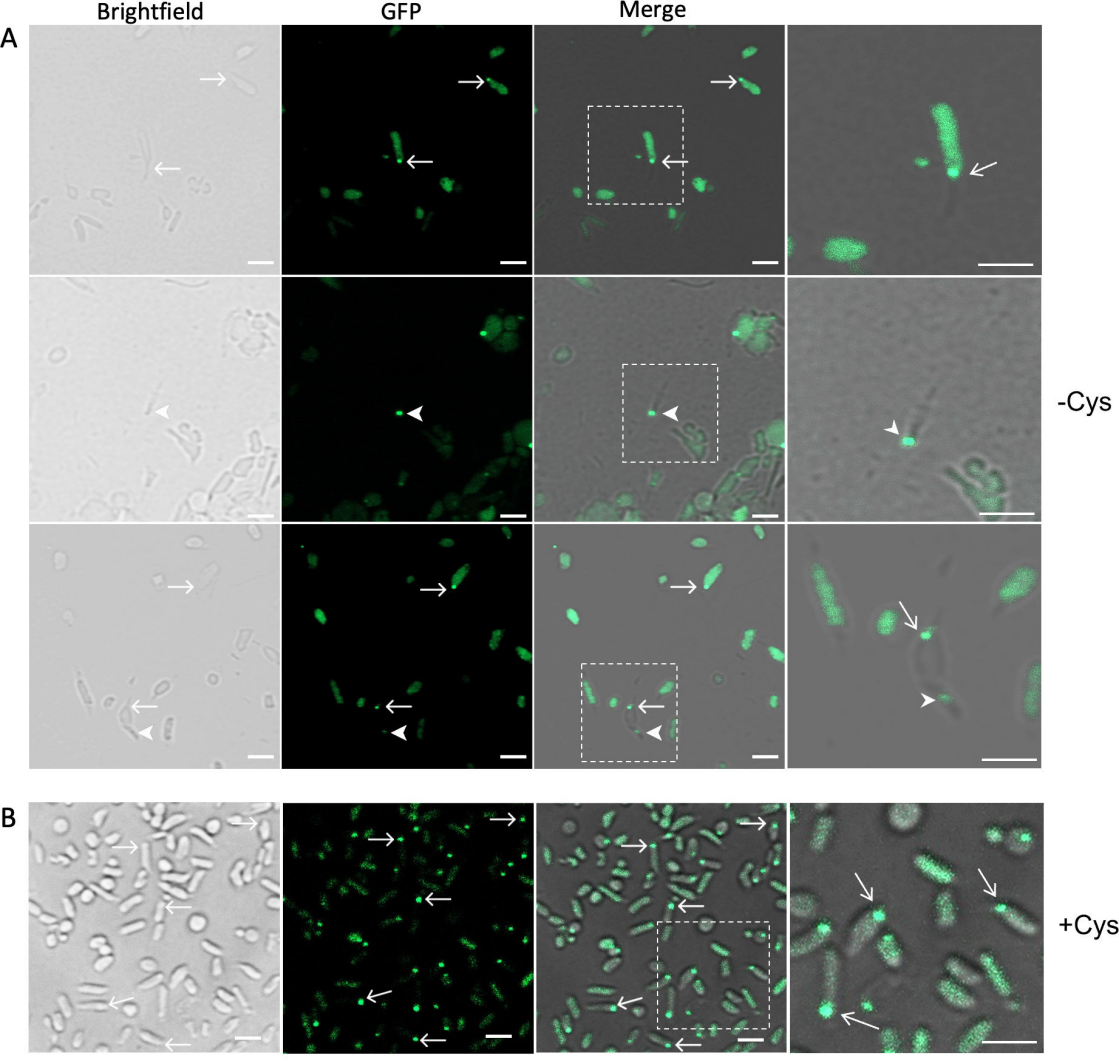
*F. novicida* T6SS co-localizes with OMT present on bacteria and released into the surrounding medium. (**A**) *F. novicida* U112 *iglA-sfGFP*, expressing the T6SS sheath protein IglA fused to GFP, was grown on BHI agar without cysteine supplementation (to induce OMT formation) and imaged by confocal microscopy. Brightfield, GFP, and merged images of the bacteria are shown. The images on the far right in each row are enlarged views of the boxed regions. The arrows point to IglA puncta located at the base of OMT extending from the bacterial surface. The arrowheads point to IglA puncta present in OMT released into the surrounding medium. (**B**) *F. novicida* U112 *iglA-sfGFP* was grown on BHI agar with 0.1% cysteine supplementation (to repress OMT formation). The arrows point to IglA puncta located at a bacterial pole. Scale bars, 2 µm.

The *Francisella* T6SS is essential for bacterial escape from the phagosome during intracellular infection. Given the observed changes to the phagosomal membrane that coincided with the presence of a bacterial OMT, we next investigated if the T6SS might localize to the tubes. To visualize the T6SS, we used an *F. novicida* U112 strain expressing a fusion of IglA with sfGFP (18). IglA forms the outer sheath of the T6SS structure (Fig. S1A) (34, 35). We grew the *iglA-sfGFP* strain on BHI agar under tube-inducing conditions (without cysteine supplementation) for 44–48 h, and single colonies were picked for visualization by confocal microscopy. By brightfield imaging, the bacteria appeared pleomorphic, as expected for bacteria undergoing tubulation. OMTs were observed extending from the surface of 37% of the bacteria analyzed (total of 90 bacteria), and released OMTs were observed in the surrounding medium (Fig. 6A). By fluorescence imaging, single, polarly localized IglA puncta were visible in 20% of the bacteria analyzed. Notably, where both IglA puncta and OMT were visible in the same bacterium (*n* = 9), the puncta always localized at the base of where the tubes extended from the bacterial surface (Fig. 6A). Thus, there was 100% co-localization of IglA with the OMT when both were visible. In addition, we observed IglA puncta within released OMT (Fig. 6A), indicating the T6SS component is exported away from the bacteria with the tubular vesicles. We also examined bacteria grown under non-tube-inducing conditions (BHI agar supplemented with 0.1% cysteine). Under these conditions, the bacteria appeared more uniform in shape, and OMTs were not observed (Fig. 6B). However, single, polarly localized IglA puncta were still present in the bacteria. Taken together, our findings reveal that the *F. novicida* T6SS localizes to sites of OMT formation and remains associated with the OMT during their maturation and release from the bacteria.

Given the observed association between the T6SS and OMT, we next tested the involvement of the T6SS in OMT production. We first used a vesicle quantitation assay to compare total OMV and OMT released by wild-type (WT) *F. novicida* U112 with *iglA*, *iglC*, and *pdpB* transposon mutants (36). IglA is the outer sheath component tagged with GFP in the above experiments; IglC forms the central tube of the T6SS; and PdpB is part of the membrane complex (Fig. S1A) (34, 35). Vesicle quantitation showed that the *pdpB* and *iglC* mutants had increased vesiculation compared to WT bacteria, whereas no difference was detected for the *iglA* mutant (Fig. S1B). To directly determine the impacts of the T6SS on OMT production, WT bacteria and the *iglC* and *pdpB* mutants were examined by negative-stain TEM. The TEM analysis showed that all bacteria produced both bacterial OMT and released tubular vesicles, with the OMT appearance comparable between the WT and mutant strains (Fig. S1C). These results demonstrate that OMT production may be modulated by but is not dependent on the T6SS.

## DISCUSSION

*Francisella* generates unique tubular extensions of its OM and releases tube-shaped vesicles into the surrounding medium (23). The structural basis for the formation of the *Francisella* OMTs and their function during infection are unknown. In this study, we characterized the OMT ultrastructure and steps leading to OMT generation in *F. novicida*. Our results revealed that major alterations in the bacterial envelope occur during tubulation and identified a dynamic cytoplasmic structure associated with OMT formation. Further analysis of *F. novicida* during macrophage infection showed that OMTs are produced within the phagosome and identified a possible association of the OMTs with the breakdown of the phagosomal membrane. This was supported by our finding that the *F. novicida* T6SS, which is required for phagosomal escape, localized to the base of the tubular projections and was present in OMT released from the bacterial surface.

Typical extracellular vesicles produced by growing gram-negative bacteria bud off from the OM and contain OM and periplasmic cargo (26, 27, 37, 38). The *F. novicida* OMTs are also formed from the OM (23). However, we reveal here that the OMTs include tubular extensions of the IM and are associated with a cytoplasmic structure that itself undergoes changes in shape and extends into the growing tube. Therefore, the OMTs are derived from both the OM and IM and contain components from both membranes, along with periplasmic and cytoplasmic material. We observed this composition for OMT extending from the bacterial surface as well as released OMT. Consistent with this, proteomic analysis of vesicles purified from *F. novicida* reported that cytoplasmic and IM proteins comprise ~40% of the total abundance of the constituent proteins (23). A variety of tubular membrane extensions have been reported for gram-negative bacteria (39–43). The *F. novicida* OMTs appear distinct from these other reported structures not only in their shape, dynamic nature, and regulation but also for the involvement of a cytoplasmic organelle in tubulation and the apparent origin of the OMT at the bacterial IM.

We detected progressive changes in the *F. novicida* cell envelope during tube initiation and maturation that occurred over a 6 h period (Fig. 4). Thus, OMT formation is driven by a defined program that is responsive to environmental cues (amino acid starvation). *F. novicida* grown in amino acid-sufficient conditions were mostly regular in shape but still exhibited notable differences in their envelope compared to canonical gram-negative bacteria, including enlarged regions of the periplasm at the cell poles and uneven sections of the IM and OM that were sometimes associated with periplasmic densities (Fig. 1). These features raise intriguing questions regarding the structural organization of the cell envelope in *Francisella* spp., such as the placement of the peptidoglycan cell wall and connections among the cell wall, IM, and OM. In addition to the unusual architecture of the cell envelope, we also observed a cytoplasmic structure associated with OMT formation and present under both amino acid-sufficient and amino acid-depleted conditions. Although its nature and composition remain to be determined, the cytoplasmic structure had the appearance of a membrane-bound organelle. The interior of the structure was clearly segregated from the bulk cytoplasm, with a lower internal density. The cytoplasmic structure was generally oval shaped and located at the cell poles (Fig. 1). The structure exhibited notable changes to a more bulb-like conformation that extended into the tubular projection as OMT formation progressed (Fig. 2). Based on its positioning at the base of the tubes and progressive changes during OMT formation, we hypothesize that the cytoplasmic structure controls tubulation by initiating extension of the IM, which then exerts outward pressure on the OM to drive OMT formation. At later stages of OMT formation, the tubular projections became narrower; the periplasmic space was more constricted; and the cytoplasmic structure assumed a fragmented appearance. These changes likely reflect steps leading to the breakdown of the tubulation machinery in preparation for OMT release.

We previously observed the production of bacterial OMT by *F. novicida* upon contact with the macrophage cell surface and within the phagosome (23). This phenomenon is not restricted to *F. novicida*, as *F. tularensis* strains also produce OMT during culture and infection of macrophages (8, 25, 28, 44). Here, we captured high-resolution images of *F. novicida* contained within the macrophage phagosome (Fig. 5), verifying that the bacteria produce OMT during infection. We did not detect released OMT during our infection studies, which may be attributed to the early time point post-infection (20 min) being insufficient to allow for OMT maturation and release from the bacteria. Alternatively, released OMTs may be present, but we were unable to capture them. Notably, in our cryo-FIB-ET imaging, where the bacterial OMT extended toward the phagosomal membrane, the membrane appeared to fragment into bleb-like structures surrounding the tip of the tube. However, additional studies are required to validate that the images indeed correspond to phagosomal membrane breakdown. Previous studies using thin-section TEM also noted vesiculation and fragmentation of the phagosomal membrane during *F. tularensis* infection (12, 28). We propose that the observed images may represent a step in bacterial escape from the phagosome into the cytoplasm. Capturing intraphagosomal *F. novicida* by cryo-FIB-ET was technically challenging, and our current analysis was limited to two such images. Therefore, future studies are needed to further dissect the role of OMT during host cell infection.

Essential for *Francisella*’s escape from the phagosome is the T6SS (14–18). The T6SS is a contractile puncturing device that injects effector proteins directly into neighboring bacterial or eukaryotic cells (34, 35). By examining *F. novicida* expressing GFP-tagged IglA, which forms the outer sheath of the contractile structure that assembles in the bacterial cytoplasm, we found that the T6SS co-localizes with the OMT (Fig. 6). IglA was observed both at the base of tubes extending from bacterial surface and contained within released OMT. In agreement with this, T6SS components have been detected by proteomics of vesicles purified from *Francisella* spp. (23–25, 44). Future studies are needed to examine T6SS activity and dynamics in the context of the OMT and host cell infection. Nevertheless, the co-localization between the T6SS, which is required for phagosomal escape, and the OMT suggests a functional association between these two elements. In contrast to other bacteria, where T6SSs are preferentially assembled along the long (narrow) axis of the cell, the *Francisella* T6SS assembles at the cell poles (18). This positioning is consistent with the polar localization observed for the bacterial OMT and reinforces the relationship between these structures. Furthermore, both the T6SS and OMT are regulated by amino acid starvation, a condition encountered by the bacteria within the macrophage phagosome (25, 31, 45, 46). We did not find evidence that T6SS is required for OMT production, which agrees with a prior genetic screen in *F. novicida* (25). However, mutations in *iglC* and *pdpB* resulted in increased vesiculation, suggesting some level of interplay between these systems. The *F. novicida* T6SS localization pattern also mirrors that of the internal cytoplasmic structure associated with OMT formation. Since the T6SS mutants still form OMT similar to WT bacteria, the cytoplasmic structure is presumably still present and functional in the mutants. Future studies will be needed to determine the relationship between the T6SS and the cytoplasmic structure. The T6SS in *Francisella* may have evolved to localize with and take advantage of the OMT to facilitate effector delivery to the host during infection. The OMT extending from the bacterial surface may facilitate contact of the T6SS with the phagosomal membrane, and the released OMT may extend the reach of the T6SS away from the bacterial surface.

In conclusion, our findings demonstrate that OMT production by *Francisella* is a regulated process involving progressive remodeling of the bacterial cell envelope under amino acid-starved conditions. Furthermore, we identify a novel cytoplasmic structure that exhibits remarkable shape alterations in tube-forming bacteria and may drive tube formation, initiating at the bacterial IM. Our observations reveal the association of OMT produced by *F. novicida* contained within the macrophage phagosome with possible alterations of the phagosomal membrane. We identified a convergence between the *Francisella* OMT and the T6SS, suggesting that the bacterial tubes and released OMT may facilitate the delivery of T6SS effectors to the phagosomal membrane, enabling bacterial escape to the cytoplasm. Although the precise role of the *Francisella* OMT in phagosomal escape and subsequent virulence requires further investigation, our study lays a foundation for understanding the molecular mechanism of bacterial tubulation and its role during *Francisella* pathogenesis.

## MATERIALS AND METHODS

### Bacterial strains and growth conditions

*F. novicida* strains U112 (BEI Resources, ATCC 15482) and U112 *iglA-sfGFP* (18) were grown in BHI medium (37 g/L BHI powder [BD Biosciences], adjusted to pH 6.8) or BHI supplemented with 0.1% cysteine. For plates, Bacto agar (BD Biosciences) was added to BHI medium at 15 g/L. Bacteria grown on plates were incubated at 37°C and 5% CO_2_. For liquid cultures, the media were incubated in the presence of 5% CO_2_ for 30 min prior to bacterial inoculation. Liquid cultures were grown at 37°C with shaking at 100 rpm. Liquid cultures were started from frozen stocks by direct inoculation, followed by overnight growth. The cultures were then diluted 1:100 to achieve an optical density at 600 nm (OD_600_) of ~0.01. Day cultures were grown until exponential phase (OD_600_ of 0.5–0.8) or stationary phase (OD_600_ of 1.2–1.4).

Transposon mutants in *iglA*, *iglC*, and *pdpB* were selected from the *F. novicida* U112 transposon mutant library (36). Mutants with transposon insertions closest to the transcriptional start site were selected for analysis. Table S1 shows the list of strains used in this study and provides the locations for the transposon insertion sites. The transposon insertion for each mutant was confirmed via PCR (36). The primers used for PCR are shown in Table S2.

### Purification of vesicles released by *F. novicida*

*F. novicida* U112 lawns from four BHI agar plates, grown for 44–48 h, were scraped and suspended in 7.5 mL of OMV buffer (20 mM HEPES, pH 7.5, 150 mM NaCl, and 0.05% sodium azide). The bacteria were centrifuged at 9,780 × *g* for 10 min at 4°C, and the supernatant was transferred to a fresh tube. The bacterial pellet was washed twice with 20 mL of OMV buffer, and the wash was added to the supernatant fraction. The collected supernatants were pooled and filtered through a 0.2 µm polyethersulfone (PES) membrane (Millipore). The filtered supernatants were then ultracentrifuged at 100,000 × *g* for 1 h at 4°C. The resulting pellet was resuspended in 500 µL of OMV buffer and stored at −20°C until analysis by cryo-EM as described in the Supplemental Material.

### Quantification of purified vesicles

To quantify purified OMV and OMT, 20 µL of the purified vesicles was diluted to 400 µL with OMV buffer. Ten microliters of 1,6-diphenyl-1,3,5-hexatriene (DPH; Invitrogen) was added at a final concentration of 200 µg/ml. Samples were incubated with DPH for 20 min. Fluorescence emitted by DPH was quantified using a PC1 photon-counting spectrofluorometer (ISS) set at 350 nm excitation and 452 nm emission wavelengths. Arbitrary fluorescence units were normalized to the total vesicle volume relative to the wet weight of bacterial cell pellets to account for any differences in growth characteristics among the WT and mutant strains.

### OMT induction assay

To generate conditioned BHI medium depleted for cysteine, overnight cultures of *F. novicida* were grown in BHI to early stationary phase (OD_600_ 1.0–1.2, ~10 h). The bacteria were removed by centrifugation at 9,780 × *g* for 10 min at 4°C, and the resulting supernatants were collected and filtered through a 0.2 µm PES membrane (Millipore) to obtain the cell-free conditioned BHI medium. Separately, day cultures of *F. novicida* were grown in 25 mL BHI (without cysteine supplementation) to early log phase (OD_600_ of 0.3–0.4, ~3 h). Bacteria were pelleted at 9,780 × *g* for 10 min at 4°C and resuspended in 25 mL of the conditioned BHI, pre-warmed for 30 min at 37°C with 5% CO_2_. The cultures were further incubated in conditioned BHI for 6 h. The bacteria were analyzed by TEM or cryo-ET (see Supplemental Material) just before resuspension in the conditioned medium and at 1 and 6 h post-resuspension.

### *F. novicida* infection of RAW 264.7 cells for TEM analysis

RAW 264.7 cells, prepared as described in the Supplemental Material, were passaged 18–24 h prior to infection to ensure 90% viability and 80% confluency at the time of infection. Macrophages were resuspended in Dulbecco’s modified Eagle medium (DMEM) with 10% fetal bovine serum (FBS) to a final concentration of 6 × 10^6^. The macrophages were pelleted (1,000 × *g* for 10 min at 4°C), and the supernatant was discarded. Day cultures of *F. novicida* were grown in BHI to early log phase (OD_600_ = 0.4), and 1.2 × 10^10^ bacterial cells were centrifuged (9,780 × *g* for 10 min at 4°C) and resuspended in 1 mL DMEM with 10% FBS. The resuspended bacteria were added to the pelleted macrophages at an MOI of 2,000. The cells were centrifuged twice (200 × *g* and 800 × *g*, 10 min each at 4°C) to facilitate contact, and the supernatants discarded. The tube containing the pelleted bacteria and macrophages was incubated in a 37°C water bath for 20 min to initiate phagocytosis. The bacteria and macrophages were then treated with 1 mL of 2.5% glutaraldehyde at 37°C for 2 min, followed by incubation on ice for 30 min. The cells were centrifuged at 10,000 × *g* for 10 min at 4°C, and the resulting pellet was resuspended in 1 mL of ice-cold phosphate-buffered saline (PBS) and processed for thin-section TEM as described in the Supplemental Material.

### Bacterial sample preparation for cryo-ET

*F. novicida* U112, grown in BHI ± cysteine, was pelleted by centrifugation at 9,780 × *g* for 10 min at 4°C, resuspended in PBS, and adjusted to an OD_600_ of 1.0. The bacterial suspension was mixed with bovine serum albumin-coated 10 nm Gold Tracer beads (Aurion), and 5 µL of the mixture was deposited onto glow-discharged cryo-EM grids (Quantifoil, Cu, 200 mesh, R2/1). The grids were set on a homemade gravity plunger and blotted at the back with filter paper for almost 4 s. Then, the grids were immediately plunge-frozen in liquid ethane and analyzed as described in the Supplemental Material.

### *F. novicida* infection of RAW 264.7 cells for cryo-FIB milling

After passaging to a new flask, RAW 246.7 cells were grown overnight on cryo-EM grids (Quantifoil, Au, 200 mesh, R1/4). Glow-discharged cryo-EM grids were treated with poly-d-lysine and washed by DMEM several times before seeding cells on the grids. For bacterial infection, *F. novicida* U112 was grown in BHI medium overnight. The overnight culture was then subcultured in fresh BHI medium until it reached an OD_600_ of 0.4. A 3 mL bacterial aliquot was pelleted by centrifugation at 2,000 × *g* for 5 min and resuspended in DMEM. Then, the RAW 246.7 culture medium was removed and replaced with the bacterial suspension. To initiate infection on the grids, co-culture samples were centrifuged at 800 × *g* for 10 min and incubated at 37°C with 5.0% CO_2_ for 20 min. The DMEM was replaced with DMEM + 10% glycerol, and the cryo-EM grids were set on a homemade plunger to prepare frozen specimens for cryo-FIB sample preparation and analysis, as described in the Supplemental Material.

### Fluorescence microscopy

*F. novicida* U112 *iglA-sfGFP* was grown on BHI agar ± 0.1% cysteine for 44–48 h. Approximately 15–20 bacterial colonies were scraped off, resuspended in 1 mL sterile PBS, and centrifuged at 9,780 × *g* for 3 min to harvest the cells. The supernatant was discarded, and the remaining cell pellet was resuspended in 500 µL PBS. Approximately 5–8 µL of resuspended cells was placed on a pad of 1% agarose in sterile PBS, followed by SlowFade Gold antifade reagent (Invitrogen), and sealed with a coverslip. Samples were imaged immediately using the Zeiss LSM 980 Airyscan 2 NLO Two-Photon Confocal Microscope.

## AFTER EPUB

*[This article was published on 19 May 2025 with an error in Huilin Li's name. The byline was corrected in the current version, posted on 21 May 2025.]*

## SUPPLEMENTAL MATERIAL

Supplemental Material mbio.01060-25-s0001.pdf Supplemental methods, table, figure, and movie legends.

Movie S1 mbio.01060-25-s0002.avi Cryo-ET of *F. novicida* U112 grown on BHI agar supplemented with 0.1% cysteine supplementation (OMT-repressing condition), corresponding to Fig. 1B.

Movie S2 mbio.01060-25-s0003.avi Cryo-ET of *F. novicida* U112 grown on BHI agar supplemented with 0.1% cysteine supplementation (OMT-repressing condition), corresponding to Fig. 1B.

Movie S3 mbio.01060-25-s0004.avi Cryo-ET of *F. novicida* U112 grown on BHI agar supplemented without cysteine supplementation (OMT-inducing condition), corresponding to Fig. 2A.

Movie S4 mbio.01060-25-s0005.avi Cryo-ET of *F. novicida* U112 grown on BHI agar supplemented without cysteine supplementation (OMT-inducing condition), corresponding to Fig. 2B.

Movie S5 mbio.01060-25-s0006.avi Cryo-ET of *F. novicida* U112 grown on BHI agar supplemented without cysteine supplementation (OMT-inducing condition), corresponding to Fig. 2D.

Movie S6 mbio.01060-25-s0007.mp4 Cryo-ET of *F. novicida* U112 within the phagosome of a RAW 264.7 cell, corresponding to Fig. 5D.

ASM does not own the copyrights to Supplemental Material that may be linked to, or accessed through, an article. The authors have granted ASM a non-exclusive, world-wide license to publish the Supplemental Material files. Please contact the corresponding author directly for reuse.
