# Supplementary material for: Outer membrane tube formation by Francisella novicida involves extensive envelope modifications and is linked with type VI secretion and alterations to the host phagosomal membrane
Source: mBio. 2025 May 19;16(6):e01060-25. doi: 10.1128/mbio.01060-25 (PMC12153307; doi:10.1128/mbio.01060-25)
Supplement: Supplemental Material — Supplemental methods, table, figure, and movie legends. [file mbio.01060-25-s0001.pdf]

SUPPLEMENTAL MATERIAL

Outer membrane tube formation by *Francisella novicida* involves extensive envelope modifications and is linked with type VI secretion and alterations to the host phagosomal membrane

Maheen Rashid, Shoichi Tachiyama, Shiwei Zhu, Hang Zhao, William D. McCaig, Jingchuan Sun, Hulin Li, Jun Liu, David G. Thanassi

SUPPLEMENTAL MATERIALS AND METHODS

TEM analysis of whole bacteria. For analysis of whole bacteria grown in liquid culture for the OMT induction assay, 1 ml of culture was centrifuged (9780 x g, 5 min, 4ºC) and resuspended in 200 µl sterile PBS. Samples were placed on polyvinyl formvar-coated copper grids (Electron Microscopy Services) and allowed to adhere for 2 min. Subsequently, the grids were treated with 1% glutaraldehyde for 1 min, washed twice with PBS, twice with water, stained with 0.5% phosphotungstic acid for 20 s, and air dried. Grids for TEM were examined using an FEI Tecnai12 BioTwinG2 electron microscope operating at an accelerating voltage of 80 kV. Images were captured using an AMT XR-60 charge-coupled device digital camera system.

Cryo-EM analysis of vesicles released by *F. novicida*. A 4 µl aliquot of the purified vesicles was applied to a freshly glow-discharged (Edwards) lacey carbon grid covered with a thin layer of continuous carbon film. After one minute incubation, excess solution on the grid was blotted with a piece of filter paper and then the grid was rapidly plunged into liquid ethane using a Vitrobot (FEI). Low dose imaging (15 e/Å) was performed in a JEM 2010F transmission electron microscope (JEOL USA) operating at high tension of 200 kV and magnification of 50,000, with an objective lens under-focus value of ~3-5 µm and with the EM grids maintained at -170°C in a Gatan 626 cryo-specimen holder. Digital micrographs were recorded on a Gatan 4K by 4K UltraScan CCD camera.

Preparation of RAW 264.7 macrophage-like cells. RAW 264.7 macrophage-like cells (ATCC TIB-71) from a cryogenic vial were thawed at 37°C in a water bath. Thawed macrophages were transferred to a 15 ml conical tube and centrifuged at 100 x g for 5 min at room temperature. After discarding the supernatant, the pellet was resuspended in 7 ml of pre-warmed DMEM (Dulbecco's modified Eagle's medium) with 10% FBS (fetal bovine serum) and transferred to a T-25 flask.

Macrophages were incubated at 37°C with 5% CO_2 and passaged twice to ensure they were actively dividing.

Thin section TEM analysis of RAW 246.7 cells infected with *F. novicida* U112. For thin section TEM analysis, infected RAW 246.7 cells were fixed in 2.5% EM grade glutaraldehyde in 0.1 M PBS, pH 7.4, for a minimum of 1 h. Subsequently, the samples were treated with 1% osmium tetroxide in 0.1 M PBS, dehydrated through a series of graded ethyl alcohol solutions, and embedded in Durcupan resin. Thin sections measuring 80 nm in thickness were sliced using a Reichert-Jung UltracutE ultramicrotome and placed onto formvar-coated slot copper grids. These sections were then stained with uranyl acetate and lead citrate. Grids for TEM were examined using an FEI Tecnai12 BioTwinG2 electron microscope operating at an accelerating voltage of 80 kV. Images were captured using an AMT XR-60 charge-coupled device digital camera system.

Cryo-focused ion beam (cryo-FIB) sample preparation. The frozen specimens of RAW 246.7 cells infected with *F. novicida* U112 were loaded into an Aquilos cryo FIB-SEM system (Thermo Fisher Scientific) maintained at around -180°C. To protect specimens from the Gallium ion beam, organic and inorganic platinum layers were coated on the entire cryo-EM grids. After finding targets using ion and electron beam images, the top parts of the RAW 246.7 cells were milled by the ion beam at 0.3 nA current. Once intracellular bacteria were visualized in SEM images, the bottom parts of the RAW 246.7 cells were milled. At ~1.0 µm sample thickness, the ion beam current was reduced to 0.1 nA and used to mill target cells to ~0.5 µm thickness. In the last step, the ion beam current was further reduced to 50 and 30 pA and used to polish lamellae to a final thickness of ~150 nm. The milling angle was 8° for all target cells. After all

lamellae were prepared, another inorganic platinum layer was coated on their surface using a shorter sputtering time.

Cryo-ET data collection and processing. The frozen specimens of *F. novicida* U112 were recorded at almost -180°C using a Titan Krios G2 300 kV transmission electron microscope (Thermo Fisher Scientific) equipped with a field emission gun, K2 detector, and a BioQuantum imaging filter (Gatan). To operate the microscope, SerialEM software (1) was used for automated acquisition of tilt series images at a magnification with a physical pixel size of 5.46 Å. The microscope stage was tilted from -45° to +45° in 3° increments using the dose-symmetric scheme in a SerialEM tilt series program. During data acquisition for cryo-FIB samples, image recording began at 8° or -8° stage angle, and tilt series images were collected from ±40° to ±56° in 3° increments using the dose-symmetric scheme in FastTomo script (2). The positive or negative tilt angles depended on the direction of lamellae in the microscope. A K3 detector (Gatan) was used to record images from the cryo-FIB samples. Motioncor2 (3) was used to correct electron beam-induced image drifts. Then, IMOD software was used to create image stacks and align images in each tilt series by tracking fiducial gold beads (4, 5). For images from cryo-FIB samples, small dots from the last sputtering step in cryo-FIB milling were used to align images in each tilt series. 4x binned images were generated by binvol command in IMOD, and then 4× binned tomograms with simultaneous iterative reconstruction technique (SIRT) were reconstructed using Tomo3D (6). 4× binned tomograms were imported to Dragonfly software (version 2022.2, Comet Technologies Canada) for 3D visualization, and then bacterial OM, IM, and oval-shaped cytoplasmic structures were segmented and pseudo colored. For ribosomes in the images, round-shaped features in the software were mapped back into the 3D images.

SUPPLEMENTAL REFERENCES

1. Mastronarde DN. 2005. Automated electron microscope tomography using robust prediction of specimen movements. J Struct Biol 152:36-51.
2. Xu A, Xu C. 2021. FastTomo: A SerialEM Script for Collecting Electron Tomography Data. bioRxiv doi:10.1101/2021.03.16.435675:2021.03.16.435675.
3. Zheng SQ, Palovcak E, Armache JP, Verba KA, Cheng Y, Agard DA. 2017. MotionCor2: anisotropic correction of beam-induced motion for improved cryo-electron microscopy. Nat Methods 14:331-332.
4. Kremer JR, Mastronarde DN, McIntosh JR. 1996. Computer visualization of three-dimensional image data using IMOD. J Struct Biol 116:71-6.
5. Mastronarde DN, Held SR. 2017. Automated tilt series alignment and tomographic reconstruction in IMOD. J Struct Biol 197:102-113.
6. Agulleiro JI, Fernandez JJ. 2015. Tomo3D 2.0--exploitation of advanced vector extensions (AVX) for 3D reconstruction. J Struct Biol 189:147-52.
7. Gallagher LA, Ramage E, Jacobs MA, Kaul R, Brittnacher M, Manoil C. 2007. A comprehensive transposon mutant library of Francisella novicida, a bioweapon surrogate. Proc Natl Acad Sci U S A 104:1009-14.

Table S1. *F. novicida* U112 transposon mutants used in this study.

Strain/plasmid	Characteristics	Position in ORF (ORF length)[a]	Source or Reference
U112	WT *F. novicida*		ATCC
iglA	U112 FTN_1324::Tn	92 (552)	(7)
iglC	U112 FTN_1322::Tn	217 (627)	(7)
pdpB	U112 FTN_1310::Tn	1289 (3279)	(7)

[a]Position of transposon insertion, in nt from start of gene.

Table S2. List of primers used in this study.

Primer name	Nucleotide sequence (5'-3')	Description
FniglA F	GCAAAAAATAAAATCCCAA	Forward primer for amplification of DNA upstream of *kan* insertion cassette
FniglA R	CACAAGAGTCTTTAATCG	Reverse primer for amplification of DNA downstream of *kan* insertion cassette
FniglC F	CGAGACCATTCATGTGAGAA	Forward primer for amplification of DNA upstream of *kan* insertion cassette
FniglC R	AGCAACTCCTTTATCTGTGC	Reverse primer for amplification of DNA downstream of *kan* insertion cassette
FnpdpB F	GCTGAAGAGGAAATATTA	Forward primer for amplification of DNA upstream of *kan* insertion cassette
FnpdpB R	CTGTAGCACATTTATATAGG	Reverse primer for amplification of DNA downstream of *kan* insertion cassette

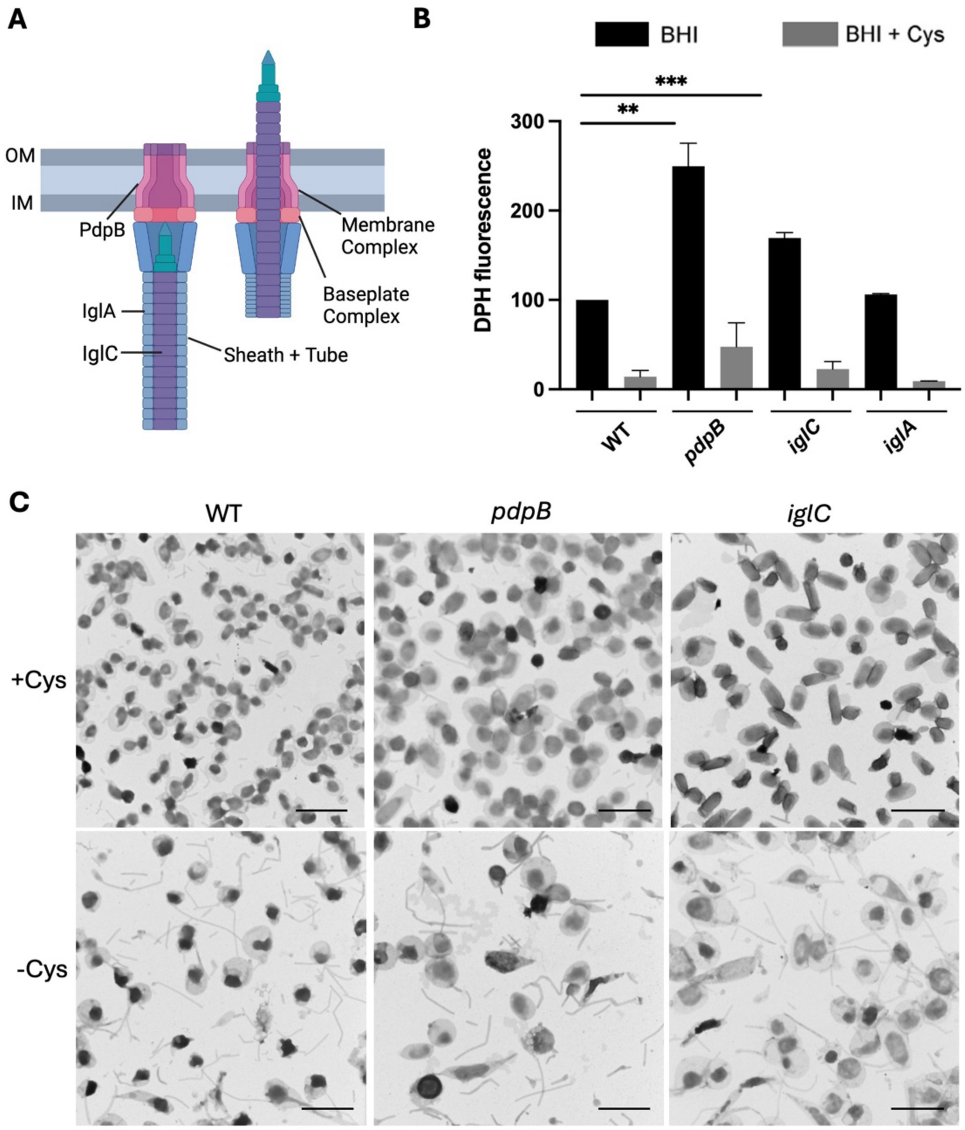

Figure S1. Vesiculation and OMT formation by *F. novicida* T6SS mutants. (A) Models of the extended (left) and contracted (right) *Francisella* T6SS. The IglA, IglC, and PdpB components are indicated. (B) Quantitation of total vesicle production by WT *F. novicida* U112 and *pdpB*, *iglA*, and *iglC* transposon mutants. The bacteria were grown on BHI agar with or without cysteine supplementation. Bars represent mean DPH fluorescence (arbitrary units) ± SEM from three independent experiments. The fluorescence was normalized to bacterial wet weight before comparing with WT bacteria grown on BHI (set at 100). **P < 0.01; ***P < 0.001 for the indicated comparisons as calculated by unpaired two-tailed Student's *t* test. (C) Representative TEM images of WT *F. novicida* and the *pdpB* and the *iglC* transposon mutants grown on BHI agar with and without cysteine supplementation. Scale bars, 2 μm.

SUPPLEMENTAL MOVIE LEGENDS

Movie S1. Cryo-ET of *F. novicida* U112 grown on BHI agar supplemented with 0.1% cysteine supplementation (OMT-repressing condition). Corresponds to the tomogram slice shown in Fig. 1A.

Movie S2. Cryo-ET of *F. novicida* U112 grown on BHI agar supplemented with 0.1% cysteine supplementation (OMT-repressing condition). Corresponds to the tomogram slice shown in Fig. 1B.

Movie S3. Cryo-ET of *F. novicida* U112 grown on BHI agar supplemented without cysteine supplementation (OMT-inducing condition). Corresponds to the tomogram slice shown in Fig. 2A.

Movie S4. Cryo-ET of *F. novicida* U112 grown on BHI agar supplemented without cysteine supplementation (OMT-inducing condition). Corresponds to the tomogram slice shown in Fig. 2B.

Movie S5. Cryo-ET of *F. novicida* U112 grown on BHI agar supplemented without cysteine supplementation (OMT-inducing condition). Corresponds to the tomogram slice shown in Fig. 2D.

Movie S6. Cryo-ET of *F. novicida* U112 within the phagosome of a RAW 264.7 cell. The tomogram is overlaid with a 3D segmentation analysis showing the bacterial IM (orange) and OM (green), and the host cell phagosomal membrane (pink). Corresponds to the tomogram slice shown in Fig. 5D.
